# Relieving nasal congestion in children with seasonal and perennial allergic rhinitis: efficacy and safety studies of mometasone furoate nasal spray

**DOI:** 10.1186/1939-4551-6-5

**Published:** 2013-03-04

**Authors:** Eli O Meltzer, Carlos E Baena-Cagnani, Davis Gates, Ariel Teper

**Affiliations:** 1 Allergy & Asthma Medical Group & Research Center, San Diego, California, USA; 2 CIMER (Research Center for Respiratory Medicine), Faculty of Medicine, Catholic University, Cordoba, Argentina; 3 Merck Sharp & Dohme Corp., Whitehouse Station, NJ, USA

**Keywords:** Mometasone furoate nasal spray, Placebo, Pediatric, Nasal congestion, Allergic rhinitis, Efficacy, Safety, Controlled clinical trial

## Abstract

**Background:**

In surveys of children with allergic rhinitis (AR), nasal congestion has been identified as the most frequently experienced and bothersome symptom. This analysis was conducted to investigate the effect of mometasone furoate nasal spray (MFNS) on congestion in children with AR.

**Methods:**

Two multicenter, double-blind, placebo-controlled studies randomly assigned children to MFNS 100 μg or placebo, 1 spray/nostril QD for 4 weeks (Study 1: ages 6–11 years with seasonal AR [SAR] ≥1 year; Study 2: ages 3–11 years with perennial AR [PAR] ≥1 year). Least square (LS) means were obtained from an ANCOVA model with treatment and study center effects, with baseline score as a covariate. We conducted post hoc evaluation of changes from baseline in AM/PM PRIOR (average of reflective AM and PM scores) nasal congestion (0=none to 3=severe).

**Results:**

Study 1: MFNS (n=134) reduced congestion significantly more than placebo (n=135) on day 2 (*P*=.004) and on 23/29 days (*P*≤.037). Change from baseline was −0.53 and −0.28 for MFNS and placebo (*P*<.001) over days 1–15 and −0.64 and −0.38 for MFNS and placebo (*P*<.001) over days 1–29. Study 2: MFNS (n=185) reduced congestion significantly more than placebo (n=189) on day 3 (*P*=.015) and on 22/29 days (*P*≤.047). Change from baseline was −0.56 and −0.36 for MFNS and placebo (*P*<.001) over days 1–15 and −0.64 and −0.45 for MFNS and placebo (*P*<.001) over days 1–29. MFNS was well tolerated, with no unusual or unexpected adverse events.

**Conclusion:**

MFNS effectively relieved nasal congestion and was well tolerated in children with SAR or PAR.

## Background

Allergic rhinitis (AR) is characterized by a range of symptoms, including nasal congestion, nasal itch, sneezing, and rhinorrhea. A large US survey of 8119 households that included 1068 children with AR indicated a 13% prevalence rate of AR [1], suggesting that millions of children in the US suffer with this condition; worldwide prevalence has been reported as high as 12% among children aged 6–7 and 22% among children aged 13–14 [2]. Nasal congestion has been identified as the most frequently experienced nasal allergy symptom by 52% of parents of pediatric patients with AR; parents of children with AR also identified congestion as the most bothersome symptom. Children with AR are predisposed to comorbidities such as otitis media, sinusitis, and asthma [1]; potential complications associated with AR-related nasal congestion in children may include sleep impairment, cognitive/emotional/behavioral disturbances, and attention deficit/hyperactivity disorder [3,4]. AR-related nasal congestion may also lead to breathing through the mouth, with a consequent risk of orthodontic abnormalities [5].

Mometasone furoate nasal spray (MFNS), a potent, topically active intranasal corticosteroid, has proven to be well tolerated and effective for the reduction and control of symptoms associated with seasonal and perennial AR (SAR and PAR); it is approved for use in children aged ≥2 years [6-8]. In particular, MFNS was shown effective, at reducing the symptom of congestion in two trials of adult patients with SAR [9], and, based on these trials and previous pediatric studies, received an FDA indication for the treatment of this symptom in patients aged ≥2 years with SAR [8]. This analysis was designed to evaluate the efficacy of MFNS in the treatment of nasal congestion in pediatric patients with SAR or PAR.

## Methods

Two multicenter, double-blind, placebo-controlled studies evaluated nasal symptom scores, including congestion, in children randomly assigned to MFNS 100 μg or placebo, 1 spray per nostril, once daily for 4 weeks. Study 1 (C95-161) was a phase 2, randomized, parallel-group, active- and placebo-controlled, double-blind study of children aged 6–11 years with ≥1 year history of SAR, with a 4-week treatment period. The study was conducted at 20 centers in the United States. Study 2 (I96-090) was a phase 3, randomized, parallel-group, placebo-controlled, double-blind, multinational study in children aged 3–11 years with PAR, with a 4-week efficacy and safety period followed by a 6-month open-label safety period (this open-label period is not included in the present analysis, as it was not placebo-controlled and could not be used to draw conclusions about symptom relief). The study was conducted at 24 medical centers in Argentina, Canada, Chile, Colombia, Finland, Guatemala, Mexico, Sweden, Uruguay, and Venezuela. Eligibility criteria for each study are shown in Table 1. In both studies, blinding of patients and investigators was maintained using placebo and active treatment identical in appearance, using a randomization schedule kept by the sponsor.

**Table 1:** Eligibility criteria

**Study 1 (SAR)**		**Study 2 (PAR)**	
**Inclusion criteria**	**Exclusion criteria**	**Inclusion criteria**	**Exclusion criteria**
Aged 6–11 years, of either sex and any race	Asthma requiring chronic use of inhaled or systemic corticosteroids	Aged 3–11 years (8–11 years in Chile and Sweden), of either sex and any race, with ≥1-year history of PAR requiring over-the-counter or prescription treatment within the year preceding the study	Asthma requiring chronic use of inhaled or systemic corticosteroids
≥1-year history of SAR that previously required treatment	Current or history of frequent, clinically significant sinusitis or chronic purulent postnasal drip	Allergic response to ≥1 clinically significant perennial allergen (house dust mite, perennial indoor mold, or animal dander prevalent in the subject’s environment) documented by a positive skin prick test (wheal diameter ≥3 mm larger than diluent control) or intradermal skin test (wheal diameter ≥7 mm larger than diluent control)	History of or current clinically significant sinus infection, chronic purulent postnasal drip, rhinitis medicamentosa, allergies to ≥2 classes of drugs, allergy to corticosteroids, or posterior subcapsular cataracts
Positive skin test response to an appropriate tree and/or grass seasonal allergen within the last year	Rhinitis medicamentosa	Nasal congestion score ≥2 (indicating a symptom of at least moderate intensity), TNSS ≥5 at both screening and baseline visits	Nasal structural abnormalities, including large nasal polyps or marked septal deviation, that significantly interfere with nasal airflow
Clinically symptomatic at both screening and baseline; nasal congestion at least moderate (score ≥2) with a total nasal symptom score ≥6	Upper respiratory tract or sinus infection that required antibiotic therapy within the previous 2 weeks, or a viral upper respiratory infection within 7 days prior to screening	Investigator-assessed overall PAR score ≥2 at baseline, and at least moderate rhinorrhea and/or congestion documented in a subject treatment diary for ≥4 of the 7 days prior to baseline	Treatment with inhaled corticosteroids for asthma for ≥2 months within the 12 months prior to enrollment or within 1 month before enrollment or 2 courses of systemic steroids, or any course lasting ≥14 days, within the 12 months preceding enrollment
	Receiving immunotherapy (desensitization therapy), unless on a stable maintenance schedule for at least 1 month prior to screening	Free of any clinically significant disease, other than AR, that could interfere with study evaluations	

AR, allergic rhinitis; PAR, perennial allergic rhinitis; SAR, seasonal allergic rhinitis; TNSS, total nasal symptom score.

**Figure 1:**
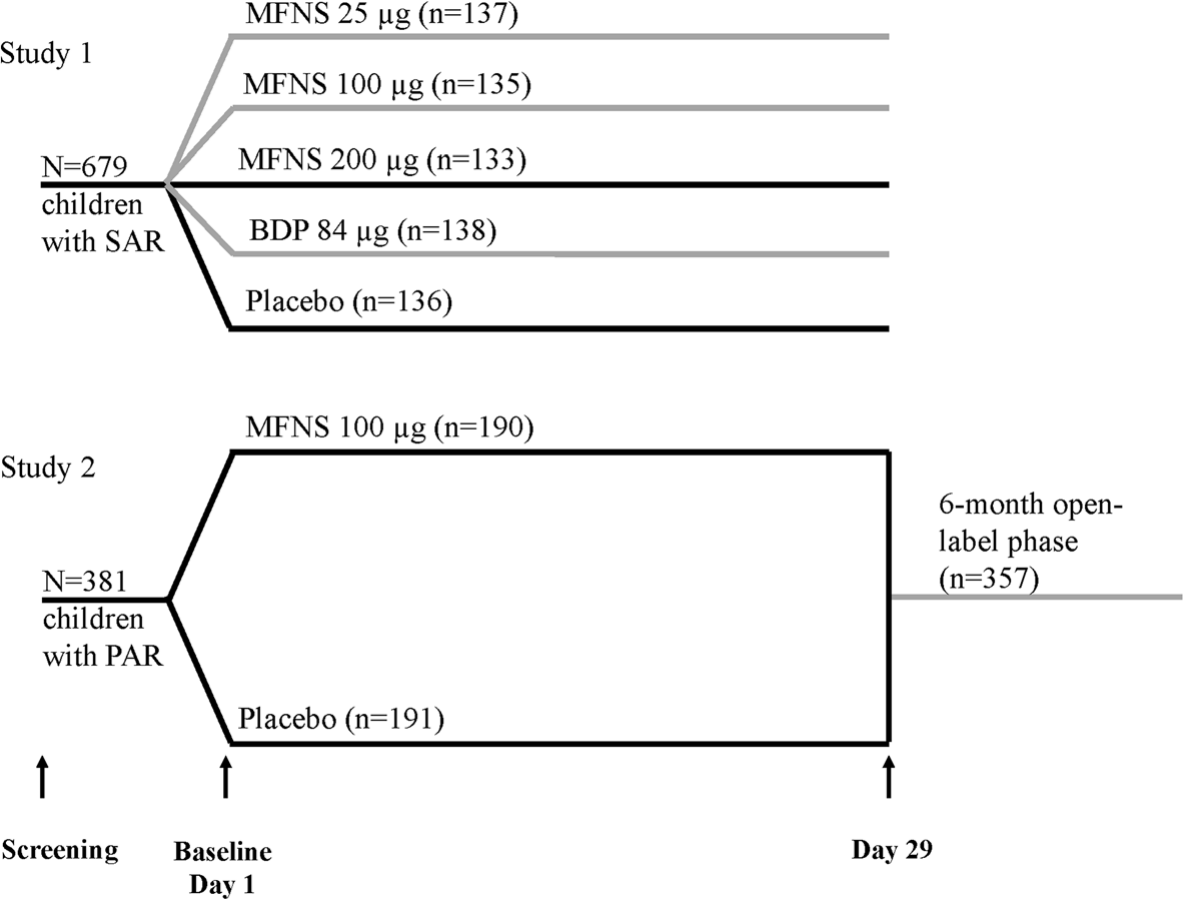
**Study designs.** BDP, beclomethasone dipropionate; MFNS, mometasone furoate nasal spray; PAR, perennial allergic rhinitis; SAR, seasonal allergic rhinitis. Gray lines indicate study arms or phases not included in this analysis of congestion results.

Both trials were conducted according to good clinical practices and the Declaration of Helsinki; an institutional review board approved the study protocol and statement of informed consent before initiation at all centers, and all patients or their guardians provided written informed consent. In Study 1, age-stratified patients were randomly assigned in a 1:1:1:1:1 ratio to treatment with MFNS 25, 100, or 200 μg once daily; beclomethasone dipropionate 84 μg twice daily; or vehicle placebo, according to a computer-generated randomization schedule. Only data from the MFNS 100 μg and placebo groups are presented in this analysis, as 100 μg is now the approved dose of MFNS in children. In Study 2, age-stratified patients were randomly assigned in a 1:1 ratio to treatment with MFNS 100 μg once daily or vehicle placebo, according to a computer-generated randomization schedule. Study designs are shown in Figure 1.

Study 1 was planned to have a sample size of 500 valid subjects, or 100 valid subjects per treatment group. This was estimated to have 90% power to detect a between-treatment difference of 1.0 points in the primary efficacy variable using a 2-sided test at an alpha level of 5% with a pooled standard deviation of 2.27 units. Study 2 was planned to have a sample size of 150 subjects per treatment group, which was estimated to have 90% power to detect a between-treatment difference of 0.85 in the primary efficacy variable using a 2-sided test at an alpha level of 5% with a pooled standard deviation of 2.27 units.

In both trials, patients and their parents or guardians rated symptoms including congestion on a scale of 0=no symptoms to 3=severe symptoms in twice-daily diaries, indicating their symptom status over the past 12 hours; physicians assessed symptoms at each visit based on the patient’s status over the previous 24 hours, up to and including the time of the current observations. In Study 1, the primary efficacy variable was change from baseline in physician-evaluated total nasal symptom score (TNSS; sum of rhinorrhea, nasal congestion, nasal itching, and sneezing) at day 8; in Study 2, the primary efficacy variable was change from baseline in physician-evaluated TNSS at day 15. For this post hoc analysis, least square (LS) mean changes from baseline congestion scores were obtained from an ANCOVA model with treatment and study center effects, with baseline score as a covariate. The software used for statistical analysis was SAS version 9.2 (SAS Institute, Inc, Cary, NC). Changes from baseline in congestion are presented according to the average of am and pm patient diary entries in each study, over days 1–15 and 1–29 and for each day.

Safety was assessed by adverse event (AE) monitoring. Additionally, in Study 1, effects on the hypothalamic-pituitary-adrenal (HPA) axis were assessed by means of cosyntropin testing at four designated study centers. Morning basal plasma cortisol level was determined, followed by injection of 0.25 mg cosyntropin; after 30 minutes, another plasma sample was drawn in which subjects were to have an increase in cortisol level of at least 7 μg/100 mL, with an absolute value >18 μg/100 mL. Plasma cortisol levels were analyzed by a 2-way ANOVA that extracted for sources of variation due to treatment, center, and treatment-by-center interaction; this analysis included differences between treatment groups before and after cosyntropin administration, along with differences between pre- and post-administration levels and change from screening in difference between pre- and post-administration levels.

## Results

In Study 1, 135 and 136 children with SAR were randomly assigned to MFNS 100 μg and placebo, respectively; in Study 2, 190 and 191 children with PAR were randomly assigned to MFNS 100 μg and placebo. Baseline characteristics are shown in Table 2. Efficacy data were available for 134 and 135 patients with SAR assigned to MFNS 100 μg and placebo, respectively, from Study 1; efficacy data were available for 185 and 189 patients assigned to MFNS 100 μg and placebo, respectively, from Study 2. Mean baseline nasal congestion score was >2 in all treatment groups, indicating that the children studied had moderate to severe obstruction.

**Table 2:** Baseline characteristics

	**Study 1 (SAR)**		**Study 2 (PAR)**	
	**MFNS 100 μg (n=135)**	**Placebo (n=136)**	**MFNS 100 μg (n=190)**	**Placebo (n=191)**
Age (years)				
Mean (95% CI)	8.7 (8.5, 9.0)	8.8 (8.6, 9.1)	7.6 (7.3, 8.0)	7.4 (7.1, 7.8)
Sex (%)				
Male	84 (62)	84 (62)	123 (65)	109 (57)
Race (%)				
White	111 (82)	113 (83)	86 (45)	89 (47)
Nonwhite	24 (18)	23 (17)	104 (55)	102 (53)
Asthma history,				
n (%)	46 (34)	62 (46)	62 (33)	62 (33)
Congestion score, LS mean (95% CI)^a^	2.20 (2.10, 2.31)	2.11 (2.00, 2.22)	2.02 (1.94, 2.10)	2.07 (1.99, 2.15)
PAR, n (%)	95 (70)	90 (66)	190 (100)	191 (100)
SAR, n (%)	135 (100)	136 (100)	52 (27)	42 (22)

^a^Scale of 0=no congestion to 3=severe congestion.

CI=, confidence interval; LS=, least squares; MFNS, mometasone furoate nasal spray; PAR, perennial allergic rhinitis; SAR, seasonal allergic rhinitis.

### Efficacy

**Figure 2:**
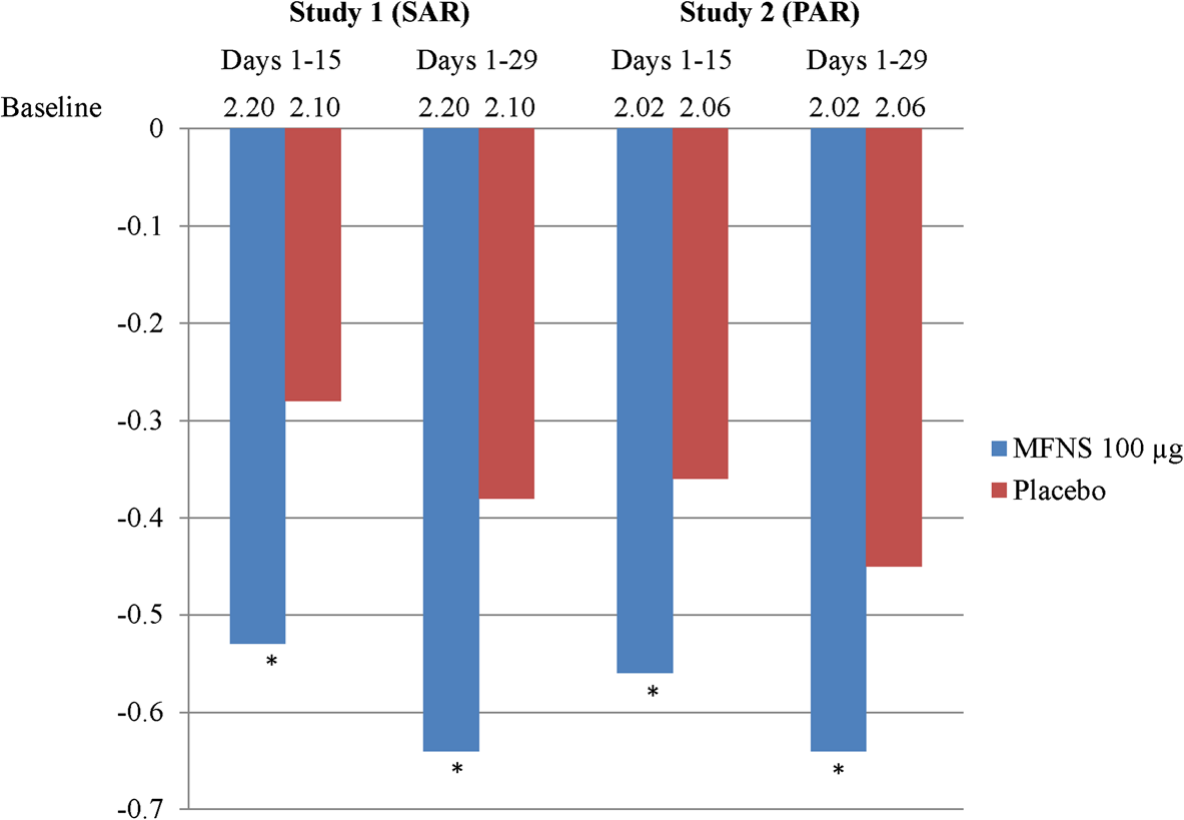
**Change from baseline congestion score over days 1–15 and 1–29.** *P<.05 vs. placebo. MFNS, mometasone furoate nasal spray; PAR, perennial allergic rhinitis; SAR, seasonal allergic rhinitis.

In both studies, MFNS was associated with significantly greater relief of congestion vs. placebo over the first half of the study and over the entire treatment period. Change from baseline over days 1–15 and 1–29 in both studies is shown in Figure 2. In the SAR study, congestion was reduced by 0.53 points (23.7%) and 0.28 points (7.8%) over days 1–15 for MFNS and placebo, respectively (*P*<.001) for a treatment difference of 0.25 points (95% confidence interval [CI], 0.12 to 0.38); over days 1–29, reductions were 0.64 points (29.4%) for MFNS vs. 0.38 points (12.1%) for placebo (*P*<.001), for a treatment difference of 0.26 points (95% CI, 0.12 to 0.40). In the PAR study, congestion was reduced by 0.56 points (22.7%) and 0.36 points (16.6%) over days 1–15 for MFNS and placebo (*P*<.001), for a treatment difference of 0.20 points (95% CI, 0.09 to 0.31); over days 1–29, reductions were 0.64 (28.2%) for MFNS vs. 0.45 (21.7%) for placebo (*P*<.001), for a treatment difference of 0.19 points (95% CI, 0.07 to 0.30). In both studies, the magnitude of treatment effect seen between days 1–15 and days 1–29 was comparable, indicating a lack of tolerance over the treatment period.

**Figure 3:**
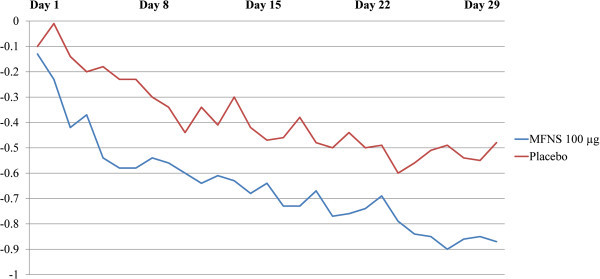
**Daily change from baseline in AM/PM congestion score, Study 1 (SAR).** Treatment difference *P*<.05 on all days except days 1, 10, 15, 18, 22, and 23. MFNS, mometasone furoate nasal spray; SAR, seasonal allergic rhinitis.

In Study 1, MFNS 100 μg was associated with significantly greater reduction of congestion vs. placebo in children with SAR, first observed on day 2 (*P*=.004) and on 23 of the 29 total days (*P*≤.037). Daily change from baseline congestion score in Study 1 is shown in Figure 3. Daily percentage reduction from baseline ranged from 1.9% on day 1 to 41.7% on day 26 for MFNS and from an increase of 1.0% on day 1 to 21.2% on day 23 for placebo. Daily treatment effect ranged from 0.03 to 0.41 points.

**Figure 4:**
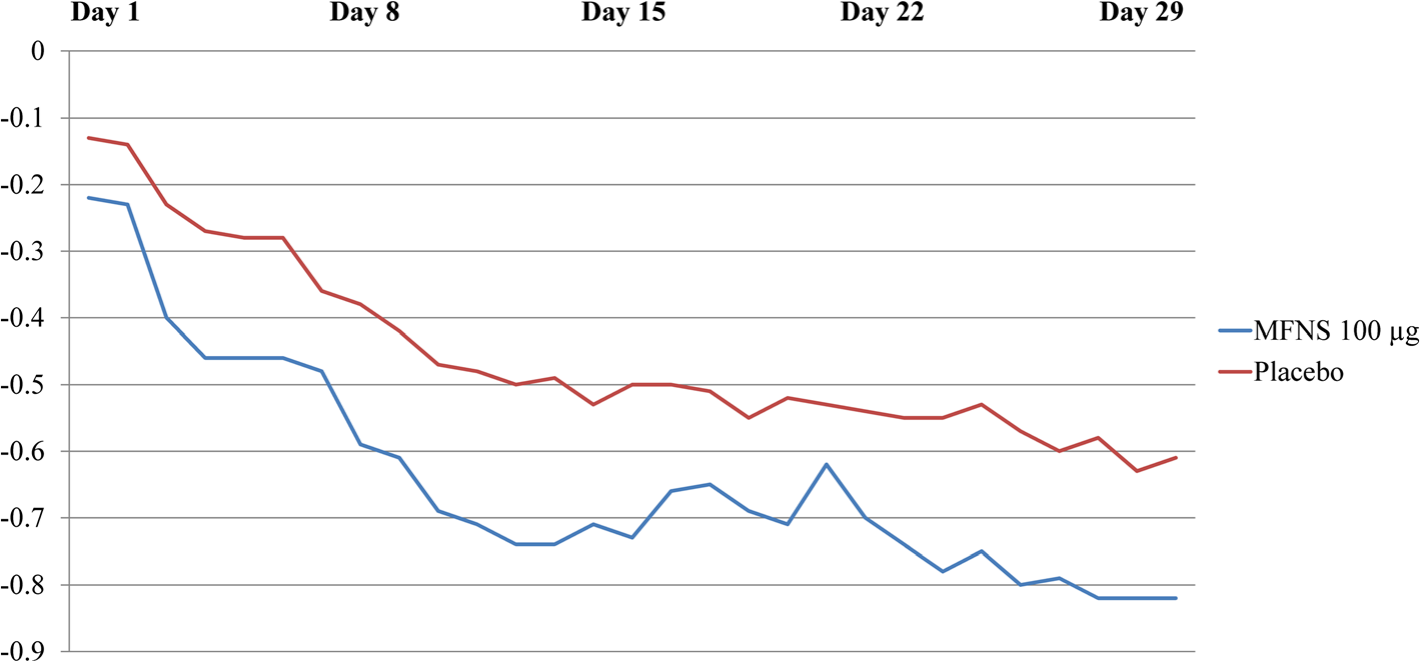
**Daily change from baseline in AM/PM congestion score, Study 2 (PAR).** Treatment difference *P*<.05 on all days except days 1, 2, 7, 17, 18, 20, and 29. MFNS, mometasone furoate nasal spray; PAR, perennial allergic rhinitis.

In Study 2, MFNS 100 μg was also associated with significantly greater congestion reduction vs. placebo in children with PAR, with significance first seen at day 3 (*P*=.015) and on 22 of the 29 total days (*P*≤.047). Daily change from baseline congestion score in Study 2 is shown in Figure 4. Daily percentage change from baseline ranged from an increase of 2.8% on day 1 to a reduction of 40.3% on day 29 for MFNS and reductions from 0.2% on day 1 to 32.0% on day 28 for placebo. Treatment difference ranged from 0.09 to 0.25 points.

As reported elsewhere, both trials met their primary end points (change from baseline in physician-evaluated TNSS at day 8 in Study 1 and at day 15 in Study 2) [6,7].

### Safety

Adverse events (AEs) were similar between treatment groups in both studies; in Study 1, treatment-emergent AEs were reported in 67% and 62% of patients receiving MFNS 100 μg and placebo, respectively, and in Study 2, treatment-emergent AEs were reported in 57% and 58% of patients receiving MFNS and placebo. Most AEs were mild or moderate in severity, and most AEs were considered by the investigator to be unrelated to treatment. Table 3 shows all AEs, all treatment-related AEs, and severe treatment-related AEs.

**Table 3:** Adverse events

	**Study 1 (SAR)**		**Study 2 (PAR)**	
**Subjects reporting AE, n (%)**	**MFNS 100 μg (n=135)**	**Placebo (n=136)**	**MFNS 100 μg (n=190)**	**Placebo (n=191)**
AEs reported by ≥5% of patients	Any, 91 (67)	Any, 93 (67)	Any, 108 (57)	Any, 110 (58)
	Fever, 9 (7)	Fever, 11 (8)	Coughing, 27 (14)	Coughing, 33 (17)
	Headache, 30 (22)	Headache, 26 (19)	Headache, 24 (13)	Headache, 25 (13)
	Vomiting, 7 (5)	Asthma, 12 (9)	Fever, 16 (8)	Fever, 15 (8)
	Asthma, 8 (6)	Coughing, 11 (8)	Pharyngitis, 14 (7)	Pharyngitis, 14 (7)
	Coughing, 7 (5)	Epistaxis, 10 (7)	Epistaxis, 12 (6)	Epistaxis, 17 (9)
	Epistaxis, 12 (9)	Pharyngitis, 15 (11)		Viral infection, 14 (7)
	Pharyngitis, 9 (7)	Sneezing, 7 (5)		
Treatment-related AEs, n (%)	Any, 27 (20)	Any, 31 (23)	Any, 28 (15)	Any, 31 (16)
	Chest pain, 1 (1)	Edema, 1 (1)	Erythema, 1 (1)	Fatigue, 1 (1)
	Fatigue, 1 (1)	Headache, 8 (6)	Fever, 1 (1)	Fever, 2 (1)
	Headache, 4 (3)	Nausea, 1 (1)	Headache, 6 (3)	Headache, 5 (3)
	Diarrhea, 1 (1)	Insomnia, 1 (1)	Malaise, 1 (1)	Crying, abnormal, 1 (1)
	Dyspepsia, 1 (1)	Somnolence, 1 (1)	Dyspepsia, 1 (1)	Dizziness, 1 (1)
	Nausea, 1 (1)	Coughing, 1 (1)	Nausea, 1 (1)	Hypokinesia, 1 (1)
	Vomiting, 1 (1)	Epistaxis, 9 (7)	Vomiting, 2 (1)	Diarrhea, 1 (1)
	Asthma aggravated, 2	Nasal burning, 2 (1)	Earache, 1 (1)	Dyspepsia, 2 (1)
	(1)	Pharyngitis, 3 (2)	Leukorrhea, 1 (1)	Nausea, 1 (1)
	Bronchitis, 1 (1)	Rhinitis, 2 (1)	Otitis media, 1 (1)	Vomiting, 1 (1)
	Coughing, 2 (1)	Sneezing, 6 (4)	Coughing, 3 (2)	Appetite increased, 1 (1)
	Dyspnea, 1 (1)	Upper respiratory tract infection, 1 (1)	Epistaxis, 7 (4)	Infection bacterial, 1 (1)
	Epistaxis, 8 (6)		Nasal burning, 1 (1)	Coughing, 5 (3)
	Nasal irritation, 3 (2)		Nasal irritation, 1 (1)	Epistaxis, 9 (5)
	Pharyngitis, 1 (1)		Pharyngitis, 2 (1)	Nasal congestion, 1 (1)
	Rhinitis, 1 (1)		Rhinorrhea, 1 (1)	Nasal irritation, 1 (1)
	Sneezing, 4 (3)		Sneezing, 5 (3)	Pharyngitis, 1 (1)
			Eczema, 1 (1)	Rhinitis, 1 (1)
			Rash, 2 (1)	Rhinorrhea, 1 (1)
			Urticaria, 1 (1)	Sneezing, 7 (4)
				Dermatitis, 1 (1)
				Rash, 1 (1)
				Urticaria, 1 (1)
				Conjunctivitis, 1 (1)
Severe treatment-related AEs, n (%)	1 (1) (headache)	4 (3) (epistaxis, rhinitis, sneezing, conjunctivitis)	1 (1) (dyspepsia)	1 (1) (nasal congestion)

AEs, adverse events; MFNS, mometasone furoate nasal spray; PAR, perennial allergic rhinitis; SAR, seasonal allergic rhinitis.

In the assessment of HPA axis effects conducted in Study 1, mean plasma cortisol levels before and after cosyntropin stimulation were similar between MFNS and placebo groups after 4 weeks of treatment (prestimulation, 10.15 [SD, 3.90] and 10.13 [SD, 3.14] μg/100 mL; poststimulation, 26.47 [SD, 3.71] and 25.95 [SD, 3.67] μg/100 mL for MFNS 100 μg and placebo, respectively). Plasma cortisol level ranges did not suggest an outlier effect.

## Discussion

In this analysis, MFNS 100 μg once daily was shown to be effective in the treatment of nasal congestion in children, whether caused by seasonal or perennial AR. Treatment effect was comparable between the study of patients with SAR and the study of patients with PAR. Though these studies were primarily designed to evaluate total nasal symptom score and not congestion score in particular, the between-treatment difference in congestion score over days 1–15 was −0.25 in the SAR study and −0.20 in the PAR study; this was comparable to the effect size of −0.23 over days 1–15 in pooled results of adult studies designed to evaluate the effect of MFNS for nasal congestion associated with SAR [9]. Results of the adult studies of the effect of MFNS on nasal congestion in SAR led to its becoming the first, and thus far the only, intranasal corticosteroid (INS) to receive FDA approval for the treatment of this symptom in patients with SAR [8].

Rather than focusing on congestion, most previous evaluations of MFNS in pediatric populations have used the total nasal symptom score as the primary efficacy variable. However, one much smaller study (N=20) evaluated the effects of MFNS on nasal congestion in children and adolescents with PAR as measured by symptom score and the objective measurement of acoustic rhinometry [10]. Mean nasal obstruction score was reduced by 50% after 21 days of treatment with MFNS 100 μg daily, and acoustic rhinometry showed significant increases in nasal volume after treatment. This small study was limited by a lack of placebo control; the present analysis found that the reduction from baseline congestion seen with mometasone was superior to that seen with vehicle placebo.

The lack of an adverse effect on the HPA axis, and overall positive safety profile, seen with MFNS in these efficacy studies correspond to the findings of prospective evaluations of the safety of MFNS in pediatric populations [11,12]. Administration of MFNS 100 μg for 1 year was found to have no effect on growth or HPA axis function per cosyntropin stimulation testing in a study of 98 children with PAR [11]. Assessment of short-term effects also found no effect of MFNS on plasma cortisol levels in children aged 6–12 years, even at a dosage of 200 μg daily for 7 days [12]. In both these studies, AE rates were similar between MFNS and placebo, as was also seen in the 2 studies examined here [11,12]. Together with the efficacy seen over the course of treatment, the safety profile of MFNS 100 μg suggests its suitability for long-term therapy in pediatric patients with AR.

This analysis indicates that baseline congestion was similar between Study 1 and Study 2. Thus, the population did not correspond to a pattern observed in other research showing PAR to be more strongly associated with nasal congestion than SAR [13,14]. However, there was considerable overlap between types of rhinitis; overall, roughly 3/5 of patients in the SAR study also had PAR, and 1/4 of the patients in the PAR study also had SAR. Other research has found similar overlap between types in a studied population [15]. The authors of the Allergic Rhinitis and its Impact on Asthma (ARIA) guidelines identify the seasonal/perennial classifications based on allergen triggers as inadequate and, rather, classify AR as “intermittent” (present <4 days a week or <4 weeks) or “persistent” (present >4 days a week and >4 weeks), although they note in their most recent guideline update that little research thus far has employed these classifications [16,17]. Despite the fact that MFNS was consistently effective in the present analysis regardless of whether AR was perennial or seasonal, it cannot be determined whether categorizing the subjects according to ARIA standards, or considering those patients who only had SAR or PAR and not the other as well, would have had any effect on the observed efficacy.

The impact and burden of AR in children (and their caregivers) has been well documented. In a survey of children and adolescents using the Child Health Questionnaire (CHQ-PF50), respondents with AR reported significantly worse results not just in general health and physical functioning but also in compromises in emotional status, mental health, self-esteem, and a number of other areas [18]. Relatively little research has been directed specifically at the symptom of nasal congestion and its impact and treatment in pediatric populations with AR. However, nasal congestion is known to be prevalent and bothersome in this population; in a survey including 500 children diagnosed with AR, nasal congestion was identified as the most frequently experienced nasal allergy symptom and was said to occur either every day (25%) or most days (27%) each week during their worst month for allergy symptoms [1]. Parents of children with AR also most frequently (27%) identified congestion as the most bothersome symptom [1]. A survey of 2355 individuals included 460 who were primary caregivers of children suffering from AR; of these, 63% identified congestion as the symptom their children most wanted to prevent and the symptom most likely cause to trigger a visit to a physician (69%) [19].

As can adults, children with AR and nasal congestion can suffer sleep disturbances, with the associated problems stemming from lack of quality sleep. A survey including 6349 children aged 5–14 years found that AR symptoms were experienced in the past year by 63.2% of habitual snorers vs 33.9% of nonsnorers (*P*<.001) [20]. Habitual snorers, in turn, were found to include a greater proportion of subjects with poor temper, hyperactivity, and poor school performance vs. nonsnorers. Children with AR-related nasal congestion can suffer sleep disturbances, including not just snoring but also restless sleep and difficulty awakening, with associated daytime sleepiness and irritability [3]. Effective treatment of congestion, including INS therapy, can provide better quality of sleep and result in better quality of life during the day. A small study (N=14) of children aged 4–9 years, all of whom had PAR with seasonal exacerbations and sleep problems such as snoring, being difficult to wake, or daytime somnolence, treated subjects with 64 μg intranasal budesonide per nostril QD for approximately 6 weeks. After treatment, subjects showed significantly improved sleep quality, as measured by change in apnea-hypopnea index (from 7.6 to 0.9, *P*=.004) and respiratory disturbance index (from 8.4 to 1.2; *P*=.005). These improvements were also reflected in quality-of-life scores related to sleep disturbance and its consequences [3]. The source data of the present analysis did not include evaluations of quality of life or sleep quality; thus, a limitation of these post hoc findings regarding the effects of MFNS on nasal congestion is that they cannot be assessed for relevance to quality of life or sleep.

The challenges of treating congestion in the pediatric population are complicated by the fact that children may not be able to articulate their symptoms to caregivers or physicians, potentially leading to missed diagnoses and undertreatment. Further, congestion related to AR presents several unique burdens in the pediatric population. Children with nasal congestion are likely to breathe through the mouth [21]. A study of 370 children aged 3–9 years, 204 of whom were chronic mouth-breathers, found that 81.4% had allergic rhinitis; children who breathed through the mouth had 78.4 times the odds of suffering nasal congestion daily [21]. Chronic mouth breathing in children is of concern, as it has the potential to affect craniofacial development [22,23]. Such possible complications, along with the fact that pediatric AR has been associated with asthma, adenoidal hypertrophy, attention deficit hyperactivity disorder, otitis media with effusion, and a 10-fold increased risk of sinus disease [1,24-27], underscore the potential adverse consequences of AR. Again, the source data of the present analysis did not incorporate evaluation of these potential comorbidities or complications and cannot be used to assess proper therapeutic management of them. Additional studies are needed on the impact of nasal congestion in children, the importance of its proper treatment, and the relationship of treatment options to complications of congestion.

## Conclusions

In this post hoc analysis, MFNS 100 μg/day demonstrated significant efficacy vs. nasal congestion in pediatric patients with both SAR and PAR, as seen in daily scores and over the course of treatment, as early as day 2 in Study 1 and day 3 in Study 2. MFNS was well tolerated in both SAR and PAR studies. These results indicate that MFNS may be a safe and effective treatment choice for children with congestion caused by seasonal and perennial AR.

## Abbreviations

AE: Adverse event; AR: Allergic rhinitis; ARIA: Allergic Rhinitis and its Impact on Asthma; HPA: Hypothalamic-pituitary-adrenal; INS: Intranasal corticosteroid; LS: Least squares; MFNS: Mometasone furoate nasal spray; PAR: Perennial allergic rhinitis; SAR: Seasonal allergic rhinitis; TNSS: Total nasal symptom score.

## Competing interests

EM reports grant/research support from Alcon, Amgen, Apotex, AstraZeneca, Boehringer Ingelheim, GlaxoSmithKline, HRA, MedImmune, Merck, Novartis, Proctor & Gamble, Sunovion, and Teva; consultant/advisory board participation for Alcon, Alexza, AstraZeneca, Bausch & Lomb, Boehringer Ingelheim, Dey, Forest, ISTA, Johnson & Johnson, Kalypsys, Meda, Merck, Mylan, ONO, OptiNose, Proctor & Gamble, Rigel, sanofi-aventis, Stallergenes, Sunovion, and Teva; and speaker participation for Alcon, ISTA, Merck, Mylan, Sunovion, and Teva. CBC reports consultancy, speaker bureau participation, and development of educational presentations for Fundación LIBRA. DG is an employee of, and AT is a former employee of, Merck Sharp & Dohme Corp., a subsidiary of Merck & Co., Inc., Whitehouse Station, NJ.

## Authors’ contributions

EM enrolled and treated patients in Study 1, contributing data generation, and contributed interpretation of the data and development of the manuscript. CBC enrolled and treated patients in Study 2, contributing data generation, and contributed interpretation of the data and development of the manuscript. DG performed the statistical analysis. AT participated in conception and design of the clinical development plan for mometasone furoate nasal spray, and helped to draft the manuscript. All authors read and approved the final manuscript.
